# Epidemiological characteristics and trends in postoperative death in children with congenital heart disease (CHD): a single-center retrospective study from 2005 to 2020

**DOI:** 10.1186/s13019-023-02224-2

**Published:** 2023-04-28

**Authors:** Guilang Zheng, Jing Wang, Peiling Chen, Zijian Huang, Lei Zhang, Aimei Yang, Jiaxing Wu, Chunlin Chen, Jingwen Zhang, Yueyu Sun, Chengbin Zhou, Haiyun Yuan, Xiaobing Liu, Jianzheng Cen, Shusheng Wen, Yuxiong Guo

**Affiliations:** 1 Department of Pediatric Intensive Care Unit, Guangdong Provincial People’s Hospital (Guangdong Academy of Medical Sciences), Southern Medical University, Guangzhou, China; 2 Guangdong Cardiovascular Institute, Guangdong Provincial People’s Hospital, Guangdong Academy of Medical Sciences, Guangzhou, China; 3 106 zhongshan Er Road, Guangzhou, Guangdong China

**Keywords:** Children, CHD; death, Thoracotomy, Epidemiological characteristics

## Abstract

**Objectives:**

To analyze the epidemiological characteristics and trends in death after thoracotomy in children with congenital heart disease (CHD).

**Methods:**

The clinical data of children with CHD aged 0–14 years who died after thoracotomy in our hospital from January 1, 2005, to December 31, 2020, were retrospectively collected to analyze the characteristics of and trends in postoperative death.

**Results:**

A total of 502 patients (365 males; 72.7%) died from January 1, 2005, to December 31, 2020, with an average of 31 deaths per year. For these patients, the median age was 2.0 months, the median length of hospital stay was 16.0 days, the median postoperative time to death was 5.0 days, and the median risk adjustment in congenital heart surgery-1 (RACHS-1) score was 3.0. 29.5% underwent emergency surgery, 16.9% had postoperative ECMO support, and 15.9% received postoperative blood purification treatment. In the past 16 years, the deaths of children with CHD under 1 year old accounted for 80.5% of all deaths among children with CHD aged 0–14 years, and deaths (349 cases) under 6 kg accounted for 69.5% of all deaths. Age at death, weight, and disease type were characterized by annual changes.

**Conclusions:**

The postoperative deaths of children with CHD mainly occurred in infants and toddlers who weighed less than 6.0 kg, and TGA and PA were the most lethal CHDs. The proportion of deaths has been increasing across the years among patients who are young, have a low body weight, and have complex cyanotic CHD.

## Introduction

Congenital heart disease (CHD) is one of the most common birth defects[1], with approximately 150,000 to 200,000 newly diagnosed cases each year and an estimated 2 million CHD patients at present in China. With advancements in medicine, especially in surgical techniques, the CHD mortality rate has decreased significantly [2–5]. In 2017, the age standardized mortality rates for CHD in China and North America were 2.63/100,000 and 1.13/100,000, respectively, decreases of 50.4% and 49.4% compared to 1990. From 1990 to 2017, CHD mortality in China decreased by 1.95% per year[6]. However, CHD remains one of the leading causes of death in Chinese children under 5-year·old[7]. Our previous research[8] suggested that the mortality rate for children with CHD in our center after surgical treatment decreased from 3.9% to 2005 to 1.1% in 2017, with total mortality showing a gradual decline. In addition, there have been many studies on CHD mortality, but there are few reports on the epidemiological characteristics of and changing trends in deaths in children with CHD after thoracotomy. Therefore, this study intends to describe the characteristics of and trends in postoperative mortality in children with CHD based on data from the past 16 years in our center.

## Materials and methods

We reviewed deaths of children with CHD after thoracotomy in our hospital between January 2005 and December 2020. Clinical data were collected from the hospital database and extracted from medical records obtained through the Medical Records Department of our hospital. The main variables analyzed were characteristics of in-hospital death and its trends from 2005 to 2020. This was a retrospective study and did not involve any risk to the children’s privacy. The study protocol was reviewed and approved by the Medical Research Ethics Committee (No. KY-Q-2021-248-01) of Guangdong Provincial People’s Hospital.

A preliminary diagnosis of CHD was determined on the basis of each patient’s symptoms and signs. Confirmation of the diagnosis was primarily obtained through cardiac color ultrasound and computed tomography. Preoperative evaluations of complicated CHD sometimes included heart 3D imaging or pulmonary angiography. Patients with severe myocardial dysfunction sometimes underwent cardiac MRI.

The inclusion criteria were ① age between 0 and 14 years, ② CHD with thoracotomy, ③ death during hospitalization. Survivors, children without CHD, children who underwent surgeries other than thoracotomy, and children older than 14 years of age were excluded. After excluding the data of 5 deaths who did not meet the inclusion criteria, the data of 502 patients were included in this retrospective study.

To compare the differences in the characteristics of cases of death in different years, the patients were divided chronologically into 3 groups: 2005–2010, 2011–2015, and 2016–2020. To investigate trends in death characteristics in different years, the characteristics of cases of death were analyzed for each year. To determine differences in disease type among pediatric patients who died in different years, CHDs were classified into 5 types according to previous studies [8, 9]. In addition, simple grouping was also performed based on the age or weight of the children.

The risk adjustment in congenital heart surgery-1 (RACHS-1) is a tool used worldwide to predict the short-term mortality and postoperative mortality due to congenital heart surgery [10]. RACHS-1 scoring was performed for patients who died. In addition, second (or more) surgery indicated that the CHD thoracotomy conducted during the last hospitalization was not the first CHD thoracotomy, and multiple rescues within 24 h were only recorded as 1 rescue. For example, the second and subsequent operations of functional single ventricular staged surgery belong to “second (or more) surgery”, while the CHD cured by the first operation does not belong to “second (or more) surgery”. Numbers of rescue shall be subject to the data recorded on the first page of the patient’s medical record. Rescue refers to the state in which the organ function of the patient losed stability and required emergency medical treatment, including cardiopulmonary resuscitation, shock, massive hemorrhage, etc. Emergency surgery refered to the operation that is urgent and needs to be operated in the shortest time, otherwise it will be life-threatening. Emergency surgery included emergency surgery for CHD and emergency surgery for postoperative complications. Low cardiac output refered to the process that cardiac insufficiency leads to the decrease of ejection fraction, resulting in insufficient oxygen supply, insufficient organ perfusion and pulmonary circulation congestion.

Statistical analyses were performed using SPSS (Statistical Package for the Social Sciences) version 19.0 (SPSS Inc). Categorical variables are described as frequency rates or percentages, and continuous variables are described as the mean, median, and interquartile range (IQR). ANOVAs was utilized while data is normally distributed, and when continuous variables were non-normally distributed, the Kruskal-Wallis test was used. Differences in percentages or proportions between groups were analyzed using the Pearson chi-square test. A two-sided p value of < 0.05 was considered indicative of statistical significance. Pie charts are used to express the composition ratio of variables, and segmented bar charts are used to visually display changes in the composition ratio of a characteristic of death in different years.

## Results

**Fig. 1:**
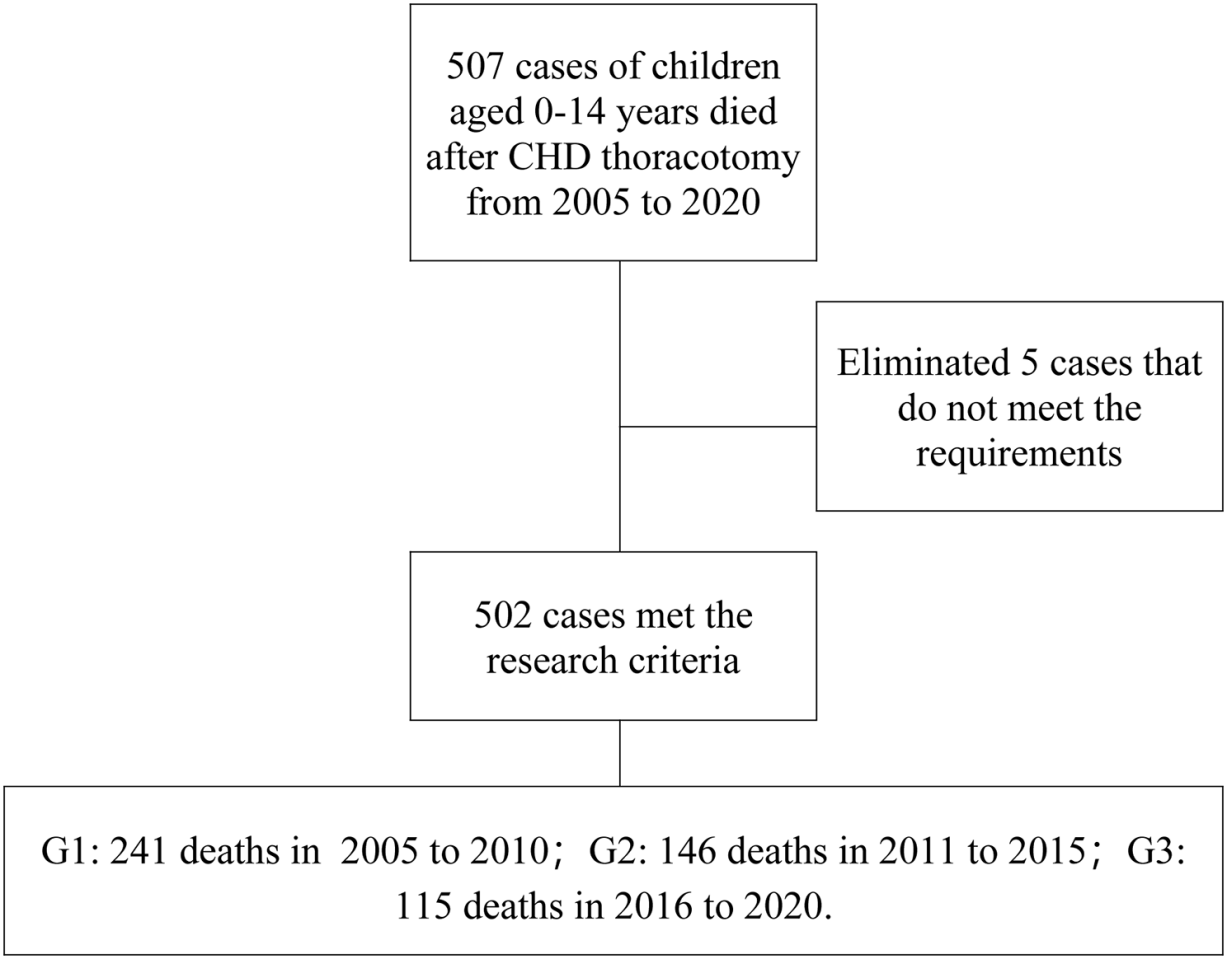
**Patient flow chart.** Reasons for exclusion were pulmonary valve stenosis with interventional surgery (1 case), congenital diaphragmatic hernia (1 case), endocardial fibrosis (1 case), congenital tracheal stenosis (1 case) and aortic valve stenosis with interventional surgery (1 case). These 5 patients did not have CHD.

Between 2005 and 2020, there were 507 cases of postoperative death of CHD patients recorded in the medical records; 5 cases did not meet the inclusion criteria and were excluded: 1 case of pulmonary valve stenosis (balloon dilatation), 1 case of congenital diaphragmatic hernia, 1 case of aortic valve stenosis (balloon dilatation), 1 case of endomyocardial fibrosis, and 1 case of congenital tracheal stenosis. The data for the remaining 502 cases that met the inclusion criteria were included in this study [Fig. 1].

Among the 502 patients who died, there were 365(72.7%) males. For these patients, the median age was 2.0 months (IQR, 0.0–9.0), and the median weight was 4.4 kg (IQR, 3.2-7.0). There were no statistically significant differences in sex, age, or body weight among the 2005–2010, 2011–2015, and 2016–2020 groups (P < 0.05). There were 167(33.3%) patients who were critically ill at admission: 104 (43.2%) patients in the 2005–2010 group, 58(39.7%) patients in the 2011–2015 group, and 5(4.3%) patients in the 2016–2020 group, with significant differences among the 3 groups (χ^2^ = 56.7, P < 0.001). The number of critically ill patients admitted to the hospital decreased across the years, suggesting that family members of patients have been more aware of CHD treatments, and the selection of elective surgery dates has been further optimized. The median length of hospital stay for all patients was 16.0 days (IQR, 9.0–29.0), and the median preoperative preparation time was 8.0 days (IQR, 5.0–13.0). There was no significant difference in the length of hospital stay or the preoperative preparation time between the 2005–2010, 2011–2015 and 2016–2020 groups (P > 0.05). The median postoperative time to death was 5.0 days (IQR, 1.0–18.0) for all patients, 3.0 days (IQR, 1.0-16.5) in the 2005–2010 group, 7.0 days (IQR, 1.0–17.0) in the 2011–2015 group, and 7.0 days (IQR, 3.0–22.0) in the 2016–2020 group, with significant differences among the 3 groups (χ^2^ = 13.3, P = 0.001). (Table 1)

**Table 1:** Characteristics of in-hospital death of CHD after surgical treatment in children

	Clinical characteristics of 502 children, (N ,% ) or median (IQR)				P value
	Total (N = 502)	2005–2010 (N = 241)	2011–2015 (N = 146)	2016-2020 （N=115）	
Age, M	2.0 (0.0–9.0)	3.0 (0.0-11.5)	3.0 (0.0-0.7.0)	1.0 (0.0–5.0)	< 0.05
Male	365, 72.7	177, 73.4	105, 71.9	83, 72.2	> 0.05
Weight, kg	4.4(3.2-7.0)	4.7 (3.2–7.5)	5.0 (3.0–7.0)	4.0 (3.0–6.0)	> 0.05
Hospital stay, d	16.0 (9.0–29.0)	16.0 (8.5–27.0)	15.5 (9.0–27.0)	19.0 (11.0–32.0)	> 0.05
Critical case on admission	167, 33.3	104, 43.2	58, 39.7	5, 4.3	< 0.01
Preoperative preparation time, d	8.0 (5.0–13.0)	9.0 (5.0–14.0)	7.0 (5.0–12.0)	8.0 (5.0–13.0)	> 0.05
Postoperative death time, d	5.0 (1.0–18.0)	3.0 (1.0-16.5)	7.0 (1.0–17.0)	7.0 (3.0–22.0)	< 0.01
s (or more) surgery	69, 13.7	9, 3.7	39, 26.7	21, 18.3	< 0.01
Number of rescues	2.0 (1.0–3.0)	2.0 (1.0–3.0)	2.0 (1.0–3.0)	3.0 (2.0–4.0)	< 0.01
RACHS-1	3.0 (2.0–4.0)	3.0 (2.0–4.0)	3.0 (3.0–4.0)	3.0 (3.0–4.0)	> 0.05
Postoperative ECMO	85, 16.9	9, 3.7	31, 21.2	45, 39.1	< 0.01
Postoperative blood purification	80, 15.9	55, 22.8	19, 13.0	6, 5.2	< 0.01
Postoperative low cardiac output	273, 54.4	124, 51.5	64, 43.8	85, 73.9	< 0.01
Emergency surgery	148, 29.5	45, 18.7	54, 37.0	49, 42.6	< 0.01

RACHS-1 is used worldwide and has been confirmed to have a strong correlation with the prognosis of congenital heart surgery. The median RACHS-1 score for all patients who died was 3.0 (IQR, 2.0–4.0). The RACHS-1 score showed an increasing but nonsignificant trend across time (χ^2^ = 3.25, P = 0.197). Among all the patients who died, 69 (13.7%) underwent second (or more) surgery: 9 (3.7%) in the 2005–2010 group, 39 (26.7%) in the 2011–2015 group, and 21 (18.3%) in the 2016–2020 group, with significant differences among the 3 groups (χ^2^ = 43.1, P < 0.001). During the hospitalization period, 148 patients (29.5%) required emergency surgery to treat the primary disease or complications: 45 patients (18.7%) in the 2005–2010 group, 54 patients (37.0%) in the 2011–2015 group, and 49 patients in the 2016–2020 group (42.6%), with significant differences among the 3 groups (χ^2^ = 27.0, P < 0.001). Eighty-five patients (16.9%) underwent extracorporeal membrane oxygenation (ECMO) support after surgery: 9 patients (3.7%) in the 2005–2010 group, 31 patients (21.2%) in the 2011–2015 group, and 45 patients (39.1%) in the 2016–2020 group, with significant differences among the 3 groups (χ^2^ = 72.1, P < 0.001). Blood purification included hemodialysis, hemofiltration, hemoperfusion, plasma exchange, peritoneal dialysis and continuous renal replacement therapy. Eighty patients (15.9%) underwent postoperative blood purification treatment: 55 patients (22.8%) in the 2005–2010 group, 19 patients (13.0%) in the 2011–2015 group, and 6 patients (5.2%) in the 2016–2020 group, with significant differences among the 3 groups (χ^2^ = 19.3, P < 0.001). The median number of postoperative rescues was 2.0 (IQR, 1.0–3.0) (Table 1).

**Fig. 2:**
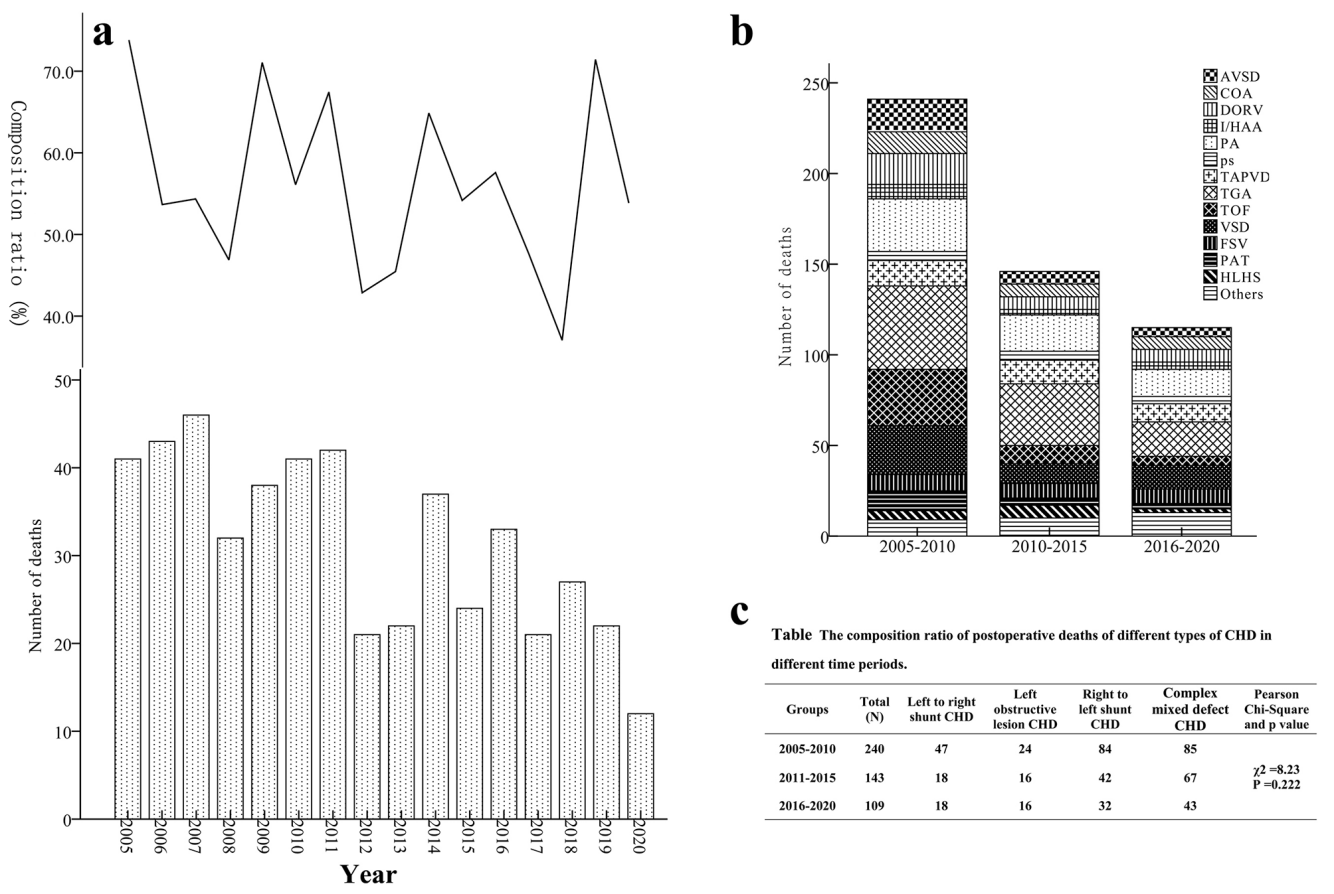
**Characteristics of Postoperative deaths of different types of CHD from 2005 to 2020.** Chart **a** shows Composition ratio of the top 7 postoperative CHD deaths (except VSD) in children from 2005 to 2020. Chart **b** demonstrates the composition of different CHD types of death in different time periods. Chart **c** shows comparison of different type CHD composition ratio of postoperative deaths in different time periods

A total of 502 patients died from January 1, 2005, to December 31, 2020, with an average of 31 deaths per year; most deaths occurred in 2007, and the least occurred in 2020. The number of deaths exhibited a downward trend year by year. In terms of disease type, the top 10 lethal CHDs were transposition of the great arteries (TGA), pulmonary atresia (PA), ventricular septal defect (VSD), tetralogy of Fallot (TOF), total anomalous pulmonary venous drainage (TAPVD), double outlet right ventricle (DORV), atrioventricular septal defect (AVSD), coarctation of the aorta (COA), functional single ventricle (FSV), and Permanent arterial stem (PAT). Although the mortality rate of left-to-right shunt CHD, including VSD, was as low as 0.69% in 2005–2017 [8], the proportion of deaths caused by left-to-right shunt CHD was still top-ranked because of the large number of VSD patients who received surgery. The total number of deaths among TGA, PA, TOF, TAPVD, DORV, and AVSD patients accounted for 57.6% of the total number of deaths between 2005 and 2020 and decreased year by year from 73.8% to 2005 to 53.8% in 2020 [Fig. 1]. The main cause for postoperative death was severe low cardiac output. The number of postoperative deaths caused by severe low cardiac output was 273 (54.4%): 124 patients (51.5%) in the 2005–2010 group, 64 patients (43.8%) in the 2011–2015 group, and 85 patients (73.9%) in the 2016–2020 group, with significant differences among the 3 groups (χ^2^ = 25.1, P < 0.001) (Table 1). To compare the chronological changes in the composition of postoperative causes of death, CHDs were classified into left-to-right shunt CHD, left obstructive lesion CHD, right-to-left shunt CHD, complex mixed defect CHD, and other CHD. Left-to-right shunt CHD, left obstructive lesion CHD, right-to-left shunt CHD, and complex mixed defect CHD were comparatively analyzed, and they were slightly but not significantly different among the 2005–2010 group, the 2011–2015 group, and the 2016–2020 group (χ^2^ = 8.23, P = 0.222) [Fig. 2].

**Fig. 3:**
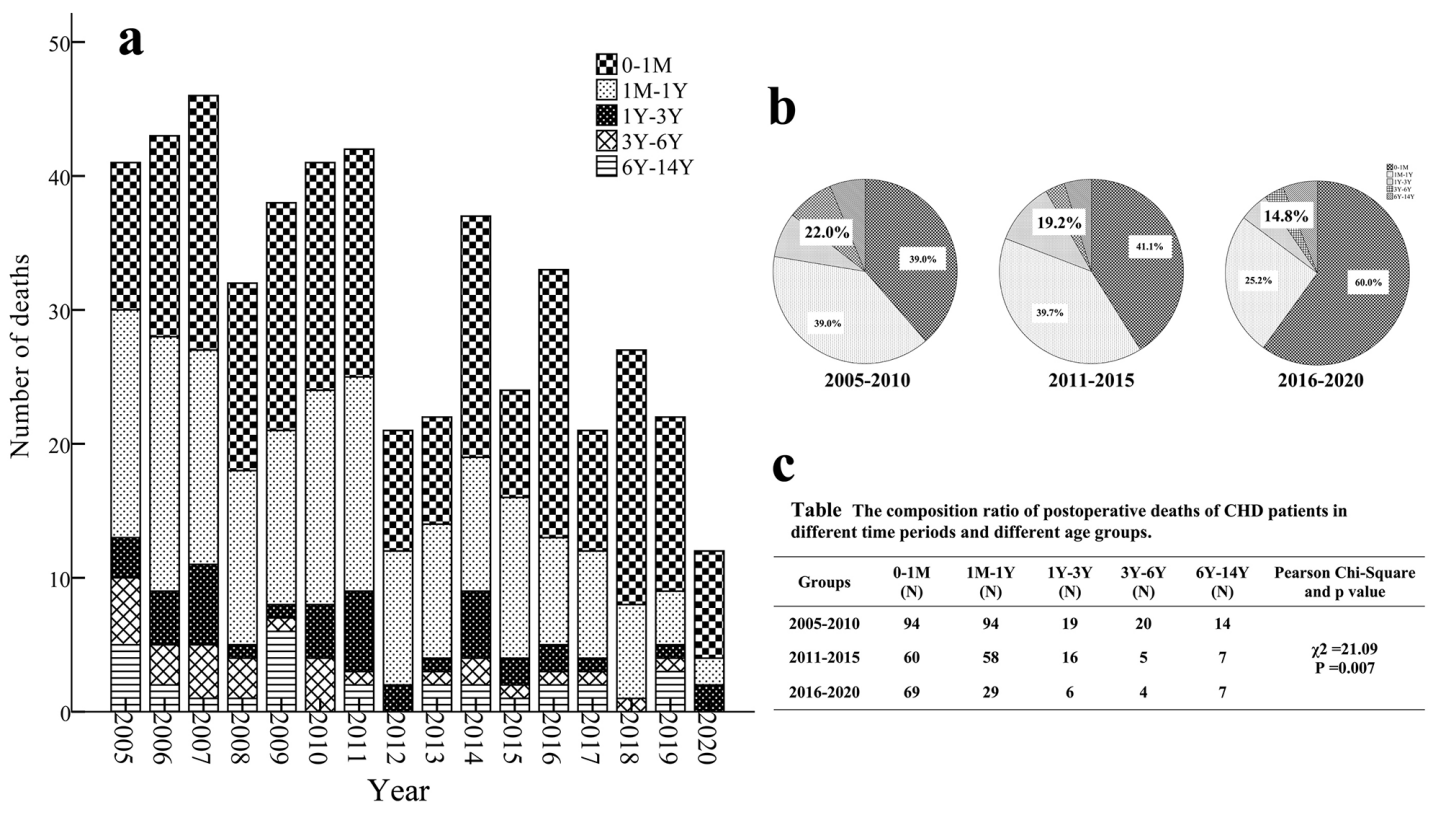
**Characteristics of postoperative deaths of CHD in different age groups from 2005 to 2020.** Chart **a** shows age composition and its changing trend of deceased children from 2005 to 2020. Chart **b** demonstrates the composition ratio of different age of death in different time periods. Chart **c** shows the difference of composition ratio of postoperative deaths of CHD patients in different age groups

The younger a patient is, the greater is the risk of death from CHD surgery. Therefore, the age composition changes and chronological changes in the patients who died were analyzed. The patients who died from 2005 to 2020 included 223 (44.4%) younger than 1 month (< 1 M group), 181 (36.1%) aged between 1 month and 1 year (1 M-1Y group), 41 (8.2%) aged between 1 year and 3 years (1Y-3Y group), 29 (5.8%) aged between 3 years and 6 years (3Y-6Y group), and 28 (5.6%) aged between 6 years and 14 years (6Y-14Y group). In the past 16 years, deaths among children with CHD younger than 1 year accounted for 80.5% of all deaths among children with CHD aged between 0 and 14 years, and the proportion of deaths among children with CHD younger than 1 year increased from 41.7% to 2005 to 84.6% in 2020. The proportions of deaths among children younger than 1 month were 39.0%, 41.1%, and 60.0% in the 2005–2010 group, the 2011–2015 group, and the 2016–2020 group, respectively. The proportions of deaths among children aged between 1 month and 1 year were 39.0%, 39.7%, and 25.2% in the 2005–2010 group, the 2011–2015 group, and the 2016–2020 group, respectively. The proportions of children aged between 1 year and 14 years in these 3 groups were 22.0%, 19.2%, and 14.8%, respectively. To further analyze the difference in age composition, the proportions of each age group in the 2005–2010 group, the 2011–2015 group and the 2016–2020 group were compared. The results showed that the age composition was significantly different among the 3 groups (χ^2^ = 21.1, P = 0.007) [Fig. 3]. Specifically, the proportion of deaths among younger children has shown an increasing trend, which indirectly reflects the reduction in postoperative deaths in older children, suggesting that treatment for CHD has been improving.

**Fig. 4:**
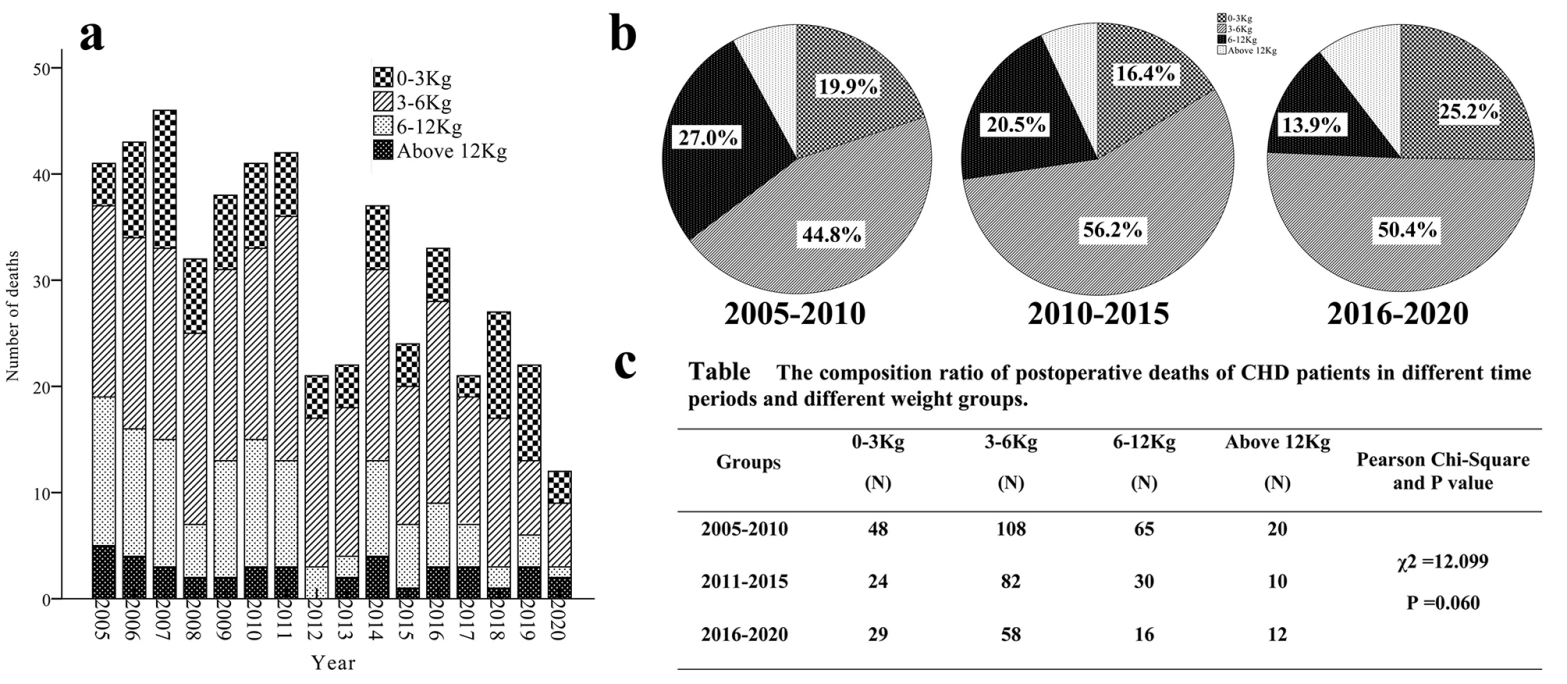
**Characteristics of postoperative deaths of CHD in different weight groups from 2005 to 2020.** Chart **a** shows weight composition and its changing trend of deceased children **from 2005 to 2020.** Chart **b** demonstrates the composition ratio of different weight of death in different time periods. Chart **c** shows the difference of composition ratio of postoperative deaths of CHD patients in different weight groups

The lower is the body weight of a patient, the more difficult is a surgery, and the higher is the risk of death. Therefore, the body weight composition and its trend across the years among the patients who died were analyzed. Among the patients who died after CHD surgery between 2005 and 2020, the proportions of patients who weighed between 0 and 3 kg (0–3 kg group), who weighed between 3 and 6 kg (3–6 kg group), who weighed between 6 and 12 kg (6–12 kg group), and who weighed above 12 kg (> 12 kg group) were 20.1%, 49.4%, 22.1%, and 8.3%, respectively, and 349 patients (69.5%) weighed less than 6 kg. From 2005 to 2020, the total proportion of patients who weighed 0–3 kg and 3–6 kg exceeded 50.0%, and 460 patients (91.7%) weighed less than 12 kg, accounting for the vast majority of patients. The proportions of patients in the 2005–2010 group, the 2011–2015 group, and the 2016–2020 group who weighed 0–3 kg were 19.9%, 16.4%, and 25.2%, respectively, and those of patients who weighed 3–6 kg were 44.8%, 56.2%, and 50.4%, respectively. Further statistical analysis indicated that there was no significant difference in the proportions of patients who weighed 0–3 kg, 3–6 kg, 6–12 kg, and greater than 12 kg among the 3 groups (χ^2^ = 12.1, P = 0.060) [Fig. 4] but that the proportion of patients who weighed below 6 kg showed an increasing trend across time. The change in the weight composition in cases of death reflected the concentration of postoperative deaths among patients with low weights and suggested improvements in the surgical treatment of CHD.

## Discussion

CHD is one of the top 5 causes of death in children under 5 years of age in China and is a serious threat to the health of children. With rapid economic development in China, the mortality rate of children under 5 years of age has decreased significantly, and the mortality rate of children with CHD under 5 years of age has also decreased significantly in both rural and urban areas [7]. From 2005 to 2020, more than 20,000 CHD children were treated in our hospital, and the mortality rate was 2.1% and showed declining trend year by year [8]. CHD is a common congenital malformation in childhood. With advancements in medical technology and surgical procedures, the CHD mortality rate is decreasing year by year [1, 5, 11, 12]. The mortality rate of surgical treatment of simple CHD, such as VSD, ASD, and patent ductus arteriosus (PDA), is lower in China than in developed countries in Europe; however, the mortality rate of complex CHD, such as PAT and left ventricular dysplasia syndrome, is significantly higher in China than in European countries [13]. In this retrospective study, 502 CHD patients died after thoracotomy, and the number of deaths each year showed a gradual downward trend from 2005 to 2020. The number of critically ill cases at admission decreased across time. With improvements in people’s living standards and their understanding of CHD, patients’ parents have the ability to assist doctors choosing, on a more scientific basis, the timing of CHD surgery, which reduces the number of patients who are hospitalized only after the disease becomes serious. The selection of elective surgery is becoming more scientific and rational.

CHD deaths are often complex, and many are associated with other malformations. RACHS-1 is widely recognized and is an important tool for predicting the short-term and postoperative mortality due to CHD surgery. The RACHS-1 scores for the patients who died from 2005 to 2020 showed an increasing trend although the difference is not statistically significant (Table 1). In addition, the proportion of patients with late-stage CHD who underwent 2 or more CHD surgeries was higher than that of patients with early-stage CHD. In recent years, increasingly more patients with substantial surgical challenges and risk have been successfully treated by surgery, suggesting improvements in surgical techniques and perioperative treatment in our hospital and indirectly reflecting improvements in people’s living standards. The surgical resources that can be used to treat complex CHD are relatively inadequate; therefore, many patients with complex CHD must wait a long time for surgery. This study indicated that the preoperative waiting time was approximately 1 week, including the perioperative preparation time. The main cause of postoperative death was severe low cardiac output syndrome within 1 week after surgery. Patients with severe CHD suffered ischemia and reperfusion injury after cardiopulmonary bypass (CPB) surgery, and ventricular or vascular dysplasia occurred in some, leading to severe low cardiac output. Our study found that the number of patients who chose ECMO support and received emergency surgery during hospitalization was significantly higher in the 2016–2020 group than in the 2005–2010 group. The exact reason is not clear but may be related to more developed techniques, the economic development level, and the perceptions of society. Ten years ago, when facing severe postoperative low cardiac output or complications that required further surgical exploration, many family members would choose to give up treatment. However, in recent years, fewer and fewer people have given up treatment.

In different years, TGA was the most common cause of death after thoracotomy in CHD patients, and TGA-caused deaths accounted for approximately 20.0% of all deaths. PA, VSD, TOF, TAPVD, DORV, AVSD, COA, FSV and PAT ranked in the top 10 lethal CHDs. The mortality rate of cyanotic CHD was higher than that of noncyanotic CHD, a finding related to the greater difficulty of surgery in patients with cyanotic CHD, the younger ages and lower weights of patients at the time of surgery, and the greater difficulty in perioperative treatment. The mortality rates of VSD/ASD/PDA, TOF, AVSD and total anomalous pulmonary venous connection in our hospital were 0.43%, 1.75%, 2.44% and 1.95%, respectively, percentages that are close to those for developed countries in Europe [13]. Due to the large number of VSD cases requiring surgery, VSD surgery accounted for the highest overall number of deaths. Compared with simple CHD, cyanotic CHD has complex intracardiac malformations and is often associated with pulmonary artery or aortic malformations. If cyanotic CHD is not treated in a timely manner by surgery, the natural fatality rate is extremely high. In addition, patients with cyanotic CHD often have very severe conditions soon after birth [14, 15]. Compared with simple CHD, cyanotic CHD is associated with more complicated perioperative management, greater surgical difficulties, more postoperative complications, and a higher mortality rate [16].

The younger are the children, the greater is the proportion of deaths after CHD surgery. This study suggested that the deaths among children younger than 1 year old accounted for the majority of all deaths, with 1-month-old children accounting for the highest proportion. Some scholars have reported [17] that the relationship between age and mortality is U-shaped and that mortality is highest among the youngest children and adults older than 60 years of age. The high mortality among young children might be related to the greater surgical challenges, higher anesthesia risk, and more complications after surgery. Studies have shown that rate of postoperative complications of surgery for neonates with CHD is as high as 29.2% and that early postoperative death may be related to body weight, Aristotle basic score, CPB time, aortic cross-clamp time, and circulatory arrest time [4]. It has also been reported [17] that factors such as age, complexity of surgery, need for emergency surgery and socioeconomic status can predict the risk of postoperative death. This retrospective survey did not analyze the risk factors for death in patients, which is undoubtedly a major limitation of this study.

The lower was the body weight of children, the higher was the proportion of postoperative deaths from CHD. This study suggested that the deaths among patients who weighed below 6 kg accounted for 69.5% of all deaths (concentrated among newborns and young infants) and that weight below 6 kg was the characteristic that accounted for the majority of all deaths in the past 16 years. In addition, the proportion of patients with medium and low weights at death has shown an increasing trend across time, suggesting that the surgical techniques for CHD and perioperative management are improving and that the deaths of CHD patients with higher weights has been decreasing. A previous small sample study in our center [18] suggested that the postoperative mortality rate for premature and low birth weight infants with CHD was 21.7%. Low birth weight infants have underdeveloped organs, poor preoperative general conditions, and a high prevalence of perioperative pneumonia. Therefore, clinical treatment for CHD is always difficult in low birth weight infants. Neonates and young infants have small myocardial cells, few contractile components, and poor ventricular compliance. Due to their low tolerance to preload and afterload, sudden circulatory failure often occurs postoperatively and leads to death [19]. Young infants have poor tolerance to CPB, and even if the anatomical deformity is completely corrected after surgery, they are still prone to multiple organ dysfunction. Therefore, strengthening perioperative management is key to reducing the operative mortality rate [20].

With the development and application of artificial intelligence, virtual reality and 3D printing, the development of CHD surgery in China has entered a new era. Further improving the treatment of critical and complex cyanotic CHD in young children with low weights should be a direction of future efforts.

## Conclusions

The results of this study suggested that postoperative deaths of infants and young children who weighed less than 6.0 kg accounted for the majority of deaths among children with CHD, that TGA and PA were the most common CHDs, and that severe low cardiac output within 1 week after surgery was the main cause of death. Deaths have concentrated among children with a young age, low weight, and complex cyanotic CHD across time. In the future, studies of children with CHD should address the diagnosis and treatment of children with a young age, a low weight, and complex cyanotic CHD and explore more and better diagnosis and treatment methods to reduce or effectively treat the severe low cardiac output syndrome that occurs 1 week after surgery.

**Abbreviation** Transposition of the great arteries (TGA), pulmonary atresia ( PA), Ventricular septal defect ( VSD), tetralogy of Fallot ( TOF), total anomalous pulmonary venous drainage ( TAPVD), double outlet right ventricle ( DROV), atrioventricular septal defect ( AVSD), coarctation of the aorta ( COA), functional single ventricle ( FSV), and Permanent arterial stem ( PTA).

## Data Availability

The datasets supporting the conclusions of this article are included within the article.

## Declarations

### Statements and Declarations

The data used in this study was anonymised before its use. The study protocol was reviewed and approved by the Medical Research Ethics Committee (No. KY-Q-2021-248-01) of Guangdong Provincial People’s Hospital. All authors stated no potential conflicts exist. All authors agree to publish the article.
